# Outcome of Children Requiring Intensive Care Following Hematopoietic Stem Cell Transplantation: A Single Center Experience

**DOI:** 10.1002/pdi3.70023

**Published:** 2025-09-27

**Authors:** Natalia Builes, Byron E. Piñeres‐Olave, Laura Niño‐Serna

**Affiliations:** ^1^ Hospital Pablo Tobón Uribe Medellín Colombia; ^2^ Clínica Somer Rionegro Colombia; ^3^ Universidad de Antioquia Medellín Colombia

**Keywords:** critical care outcomes, hematopoietic stem cell transplantation, mechanical ventilation, mortality, pediatric intensive care unit, pediatrics

## Abstract

Hematopoietic stem cell transplant (HSCT) is associated with some complications requiring advanced support in the pediatric intensive care unit (PICU). However, the outcome of children requiring admission to a PICU following HSCT in middle‐income countries (MICs) are limited. One hundred and four children undergoing hematopoietic stem cell transplantation at a bone marrow transplant service in Colombia from January 2012 to June 2019 were enrolled. Baseline characteristics and clinical courses were described. In addition, we compared some characteristics of patients who survived or died in the PICU through a bivariate analysis. Twenty five PICU admissions were reported. Sixty‐four percent survived to be discharged from any PICU admission. Immunodeficiency was the most common underlying disease among patients admitted to the PICU (33%). Respiratory problems (12/25, 48%), and cardiovascular instability (10/25, 40%) were the most common reasons for admission. Cardiovascular support was the most common PICU treatment (21/25, 84%), followed by respiratory support (18/25, 72%). We found that children who require admission to PICU after an allogeneic hematopoietic stem‐cell transplantation (Allo‐HSCT) present a high mortality rate. Invasive respiratory support, higher vasoactive‐inotropic score, renal replacement therapy, and the presence of multi‐organ failure were associated with mortality.

## Graphical abstract

Children who require admission to PICU after an Allo‐HSCT present a *high mortality rate*. Invasive respiratory support, higher vasoactive‐inotropic score, renal replacement therapy, and the presence of multi‐organ failure are associated with mortality. (More details of two Figures are in Figure 1 and 2).

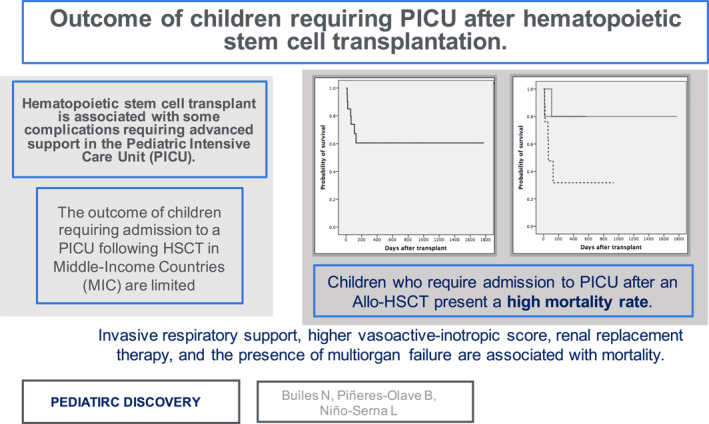

## 1 Introduction

Hematopoietic stem cell transplant (HSCT) is a potentially curative therapy in an increasing number of malignant and nonmalignant conditions in the pediatric population [1]. Advances in HSCT techniques, better recognition, and management of post‐transplant complications and infections have improved the outcome of these patients in recent years. However, HSCT is linked to some complications and major morbidity requires advanced support in the pediatric intensive care unit (PICU) in certain patients. Available data suggest that approximately one‐third of pediatric HSCT recipients will require admission to the PICU and among those requiring PICU admission, the mortality rate is high, around 36%–74%, with higher mortality rates in those receiving mechanical ventilation [2, 3, 4].

Looking at mortality rates over time, the overall trend has been improving since the earliest papers, where mortality rates were reported to be greater than 80% [5]. Nowadays, critical care medicine has evolved dramatically with an increasing focus on lung‐protective ventilation techniques, early and aggressive goal‐directed therapy in sepsis, and a wide range of renal replacement therapy (RRT) modalities [3]. All these improvements aim at addressing important factors associated with mortality in PICU patients.

The main motivation for our research is the historical lack of data describing the outcome of pediatric patients requiring admission to a PICU following HSCT in middle‐income countries (MICs). Among different reasons, most of the studies have been carried out in developed nations with an underrepresentation of developing countries and specifically the Latin American ones. We performed a retrospective study to review all allogeneic hematopoietic stem‐cell transplantation (Allo‐HSCT) recipients admitted to PICU over a 7‐year period to determine whether our mortality rates were comparable with those reported in previous studies. In addition, we report the outcome in terms of survival and search for factors that can predict the outcome once a child has been admitted to PICU in our institution. Finally, provide the possibility to have a different perspective on what has been addressed and achieve new questions to be answered in future prospective studies.

## 2 Materials and Methods

We conducted a retrospective cohort study. The study cohort included all patients up to 16 years of age at the time of Allo‐HSCT between January 1, 2012, and June 3, 2019. During the study period, our PICU did not have an institutionalized admission policy, and the appropriateness of a patient admission was determined on a case‐by‐case basis after consultation and discussion between the referral and accepting physicians. The study was approved by the Institutional Ethics Committee of the Pablo Tobón Uribe hospital in accordance with the Declaration of Helsinki (approval number: 2018.074).

Data collected included general demographic data, underlying diseases, transplant type, donor source, and conditioning regimen. Myeloablative conditioning regimens (MACs) refer to the administration of total body irradiation (TBI) and/or alkylating agents at doses which will not allow autologous hematologic recovery. Reduced intensity conditioning (RIC) regimens, which are defined as regimens containing reduced doses of myeloablative drugs (or radiotherapy), are therefore less likely to achieve marrow ablation. Two different disease categories were defined: malignant (acute leukemia, juvenile myelomonocytic leukemia [JMML], chronic myeloid leukemia [CML], dendritic tumor cells, and Hodgkin lymphoma) and nonmalignant conditions (metabolic diseases, immunodeficiencies, bone marrow failure syndrome, histiocytic disease, acquired aplastic anemia, and hemoglobinopathies). In addition, transplant characteristics were collected. The PICU variables collected were data regarding the reason for admission, timing to PICU admission, and intensive‐care treatment received. Reasons for admission were grouped as respiratory compromise, developing severe dyspnea, or other clinical signs of respiratory distress requiring some type of ventilatory support; cardiovascular instability (shock and/or hypotension requiring inotropic support); neurological compromise, major deterioration in consciousness with or without seizures; and proven infection at the time of admission was also registered.

We listed patients according to various conditions or interventions we wished to analyze. The severity of illness measures has long been used in pediatric critical care. The Pediatric Risk of Mortality (PRISM) scale is a physiologically based score used to quantify physiologic status, and when combined with other independent variables, it can compute expected mortality risk and expected morbidity risk in the first 24 h after being admitted to the PICU. For our study, we used PRISM, the endorsed and authorized scale in our hospital [6]. High‐frequency oscillatory ventilation (HFOV), which has a recommendation for patients with persistent and refractory hypoxia, as well as patients with severe ventilation disorders, is a “rescue” therapy for patients with severe oxygenation disorders and patients with moderate to severe pediatric acute respiratory distress syndrome (PARDS), both of which are associated with fatal outcomes given the degree of molecular dysfunction [7]. Moreover, given the severity of a patient's myocardial dysfunction or shock, it has been shown that the more drugs the patient needs, and in higher doses, the greater their chance of dying; to measure this, the vasoactive‐inotropic score (VIS) were recorded [8]. A frequently compromised organ is the kidney, leading the patient to renal injury and with it, oliguria and inefficiency in nitrogen clearance, force some type of dialysis therapy. In patients with hemodynamic instability, the most widely used and recommended dialysis therapy is continuous RRT, which has efficient clearance as well as fluid elimination, minimizing fluid overload in these patients; this type of therapy is also associated with increased mortality in critically ill patients [9, 10]. Finally, multiple organ failures (MOF), defined by the failure of at least 2 organ systems, was also analyzed [11]. For those patients who did not survive, the day of death before or after graft infusion and the cause of death were documented.

A descriptive statistical analysis was conducted for all selected variables and subgroups. Categorical variables were expressed as frequencies and percentages, whereas continuous variables were summarized using either the mean (standard deviation) or median (interquartile range [IQR]), depending on the distribution of the data. The normality of the data was assessed using the Shapiro–Wilk test, and transformations were applied where appropriate to meet assumptions of normality. To compare characteristics between patients who survived or died in the PICU, we performed the following statistical tests: Chi‐square test or Fisher's exact test, which is used for comparing categorical variables; Mann–Whitney *U* test, which is used for continuous variables that were not normally distributed. For survival analysis, we utilized the Kaplan–Meier method to estimate survival curves, and the Log‐Rank test was used to compare survival between different transplant types. The Cox proportional hazards model was employed to assess the independent association of various clinical and demographic variables with mortality. Hazard ratios (HRs) and 95% confidence intervals (CIs) were calculated for each variable included in the model. Multivariable adjustments were made for confounding variables as appropriate. All statistical analyses were conducted using SPSS version 20.0 (IBM Corp., Armonk, NY, USA). A *p*‐value of less than 0.05 was considered statistically significant.

## 3 Results

### 3.1 Characteristics of Children Undergoing HSCT

A total of 104 (62 males) patients received 110 Allo‐BMT during the study period. The median age at transplant was 7 years (range, 1 month–16 years). The most common underlying diagnosis was hematologic malignancy in 67/104 patients (64%). Overall survival (OS) was 57%. The main causes of death were disease relapse in 20/45 patients (44%), infection in 17/45 patients (38%), graft‐versus‐host disease in 5/45 (11%) and disease progression in 2/45 patients (4%). Demographic and transplant‐related characteristics are shown in Table 1.

**TABLE 1:** Demographics and transplant‐related characteristics.

Variable	All children (*n* = 104)	PICU admissions (*n* = 21)	Long‐term survivors from PICU (*n* = 14)
Median age (years)	7	4	6
Diagnosis, *n* (%)			
ALL/AML	60 (57)	6 (29)	5 (36)
Immune deficiencies	19 (18)	7 (33)	4 (29)
Bone marrow failure syndrome	6 (6)	3 (14)	1 (7)
Hemoglobinopathies	5 (5)	3 (14)	2 (14)
Metabolic disorders	5 (5)	2 (10)	2 (14)
Juvenile myelomonocytic leukemia	4 (4)	—	—
CML	3 (3)	—	—
Other malignant hematological	2 (2)	—	—
Donor type *n* (%)			
Haplo‐identical	52 (50)	7 (33)	6 (42)
MSD	27 (26)	5 (24)	4 (29)
Cord blood	25 (24)	9 (43)	4 (29)
Conditioning regimen, *n* (%)			
Myeloablative conditioning (MAC)	68 (65)	15 (71)	10 (71)
Reduced intensity conditioning (RIC)	36 (35)	6 (29)	4 (29)
GvHD prophylaxis, *n* (%)			
CNI ± MTX	73 (70)	15 (71)	9 (64)
CNI ± MMF	29 (28)	4 (19)	4 (29)
Cyclosporine alone	2 (2)	2 (10)	1 (7)
Serotherapy, *n* (%)	35 (34)	12 (57)	7 (50)

Abbreviations: ALL: acute lymphoid leukemia, AML: acute myeloid leukemia, CML: chronic myelogenous leukemia, CNI: calcineurin inhibitors, GvHD: graft‐versus‐host disease, MMF: Mycophenolate mofetil, MSD: Matched sibling donor, MTX: Methotrexate.

### 3.2 Characteristics of Children Admitted to the PICU

Among the 104 patients who underwent Allo‐HSCT in the study period, 21 (20%) were admitted to the PICU. Seventeen of 21 (81%) patients were admitted once, whereas four patients required a second admission. Of the six patients who received a second transplant, two (33%) were admitted to the PICU. In the PICU cohort, immunodeficiency was the underlying cause in the majority of patients, reported 7/21 (33%). There was no significant difference in age (*p* = 0.2), gender (*p* = 0.4), donor type (*p* = 0.06), and conditioning type (myeloablative, reduced intensity, serotherapy, *p* = 0.5) for those admitted to PICU compared to those who were not.

### 3.3 Reason for Admission

Of all 25 admissions, 12 (48%) were admitted due to respiratory conditions, 10 cases (40%) were related to cardiovascular instability, and 3 (12%) cases had neurological ailments (encephalopathy). Respiratory conditions included respiratory failure, PARDS due to pneumonia, and a massive pulmonary embolism. Cardiovascular instability included septic shock in 8/10 patients (80%) and hypovolemic shock in 2/10 (20%). One of these patients suffered a cardiorespiratory arrest. Fifteen of 25 (60%) PICU admissions had a proven infection (isolated microorganism) at the moment of the admission. The most prevalent microorganisms isolated were *Enterobacter cloacae* (2 patients), *Staphylococcus aureus* (2 patients), and *Aspergillus* (2 patients). Other bacteria were *Staphylococcus epidermidis*, *Pseudomonas aeruginosa*, *Listeria monocytogenes*, *Enterococcus faecalis*, *Rothia mucilaginosa*, and *Streptococcus pneumoniae*. The virus isolated was *Rotavirus* and *Cytomegalovirus*. One patient had *Pneumocystis jirovecii*. Mortality occurred in 6 of 15 patients with proven infection (40%) and in 3 of 10 patients without infection (30%); this difference was not statistically significant (*p* = 0.6).

Regarding the specific complications related to the transplant, eight patients (32%) experienced acute graft‐versus‐host disease (GvHD). In three patients, the gastrointestinal system was compromised, the skin in two, and the liver in only one. The three organs were involved in three people, and the gastrointestinal tract and skin were involved in one patient. Four patients had chronic GvHD, 16%. Three patients experienced veno‐occlusive disease (12%). Thrombotic microangiopathy was not reported in our cohort.

### 3.4 Timing and Duration of Admission

The median PICU stay for all episodes was 8 days (IQR 3.5–15). The median PICU stay for the first admission was 8 days (IQR 4–15) and for the second admission was 7.5 days (IQR 1–19). The timing of PICU first admission in the post‐HSCT period was 14 days (range −6–76) after transplantation. There was no significant difference between the time of transplantation and the admission to the PICU for survivors and non‐survivors Table 2. The median hospital stay was 44 days (IQR 30–69) for all patients admitted to the PICU; the median stay for survivors was 32 (IQR 25–95) and 47 (IQR 30–53) for non‐survivors. Two patients were admitted during the conditioning stage.

**TABLE 2:** Comparison for clinical variables between survivors and non‐survivors from PICU.

Variable	All admissions *n* = 25	Non‐survivors *n* = 9	Survivors *n* = 16	*p*‐value
Age (median, years)^c^	4	4	6	0.6^b^
Diagnosis, *n* (%)^c^				
Malignant	6 (29)	1 (14)	5 (36)	0.6
Nonmalignant	15 (71)	6 (86)	9 (64)	
Conditioning, *n* (%)^c^				
MAC	15 (71)	5 (71)	10 (71)	> 0.9
RIC	6 (29)	2 (29)	4 (29)	
Days post‐HSCT at admission (median)	16	14	16.5	> 0.9
CVC At admission, *n* (%)	16 (64)	5 (56)	11 (69)	0.6
Noninvasive ventilation, *n* (%)	4 (16)	2 (22)	2 (13)	0.6
Mechanical ventilation, *n* (%)	17 (68)	9 (100)	8 (50)	0.02
Duration of MV (median, day)	17	13	7.5	0.4^b^
HFOV, *n* (%)	10 (40)	6 (67)	4 (25)	0.08^a^
Vasoactive agent, *n* (%)	21 (84)	9 (100)	12 (75)	0.2^a^
Vasoactive inotropic scores (median)	13	50	8.5	0.03
< 30, *n* (%)	17 (68)	3 (33)	14 (88)	0.01^a^
> 30, *n* (%)	8 (32)	6 (67)	2 (13)	
Renal replacement therapy, *n* (%)	5 (20)	4 (44)	1 (6)	0.04
Multi‐organ failure, *n* (%)	12 (48)	8 (89)	4 (25)	0.004^a^
PRISM (median of percentage risk of dying, [%])	19	46	10.5	0.1
Length of PICU (median, day)	8	14	6.5	0.5^b^

Abbreviations: CVC: central venous catheter, HFOV: High‐frequency oscillatory ventilation, HSCT: hematopoietic stem cell transplantation, MAC: Myeloablative conditioning, MV: mechanical ventilation, RIC: reduced‐intensity conditioning.

^a^ Fisher's exact test.

^b^ Mann–Whitney *U*‐test.

^c^ Variables analyzed in 21 patients.

### 3.5 PICU Treatment

Of the 25 episodes that were analyzed, 18 (72%) required respiratory support, 17 (68%) mechanical ventilation (MV), and 4 (16%) noninvasive ventilation (NIV). Ten patients (40%) needed HFOV, 21 (84%) vasoactive, and 5 (20%) needed some type of RRT. Seventeen patients (68%) required both respiratory and vasopressor support. Five patients (20%) required RRT in addition to hemodynamic and ventilatory support. Four patients (16%) did not require any intensive care intervention. They were transferred for monitoring secondary to an imminent risk of clinical deterioration. There was a significant difference in terms of mortality in the following categories: the need for MV, RRT, higher VIS, and the presence of MOF (Table 2). Other PICU characteristics between survivors and non‐survivors are described in Table 2.

### 3.6 Outcome

**FIGURE 1:**
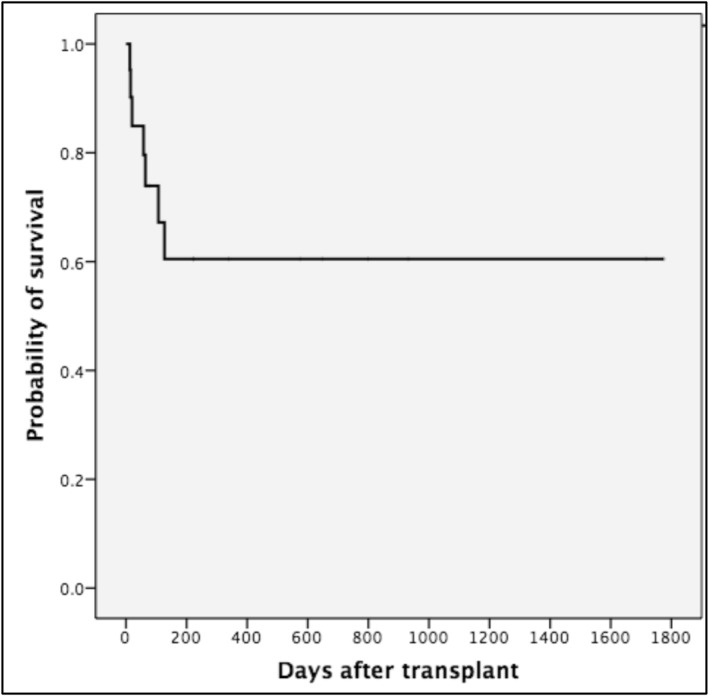
Kaplan–Meier survival plot of patients first admitted to PICU post‐HSCT (21 children).

**FIGURE 2:**
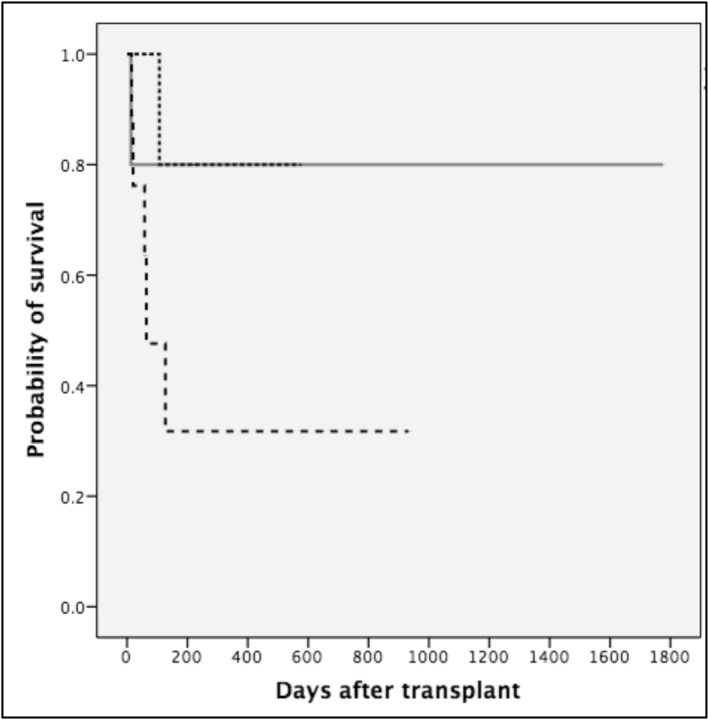
Kaplan–Meier survival plot by transplant type: patients admitted to the PICU post‐HSCT (21 children). Dotted black line: haploidentical; continuous gray line: matched related donor; discontinuous black line: UBC.

Of the 25 admissions to the PICU, 16 patients (64%) survived, 14/21 in the first admission (66%), and 2/4 (50%) in the second (Figure 1). In comparison, 52/83 (63%) patients who were never admitted to PICU survived. There was no significant difference in terms of mortality among the different transplant types for those patients admitted to PICU (*p* = 0.1, Figure 2). The main cause of death in patients admitted to the PICU was an infection in 8/9 (89%), and one patient (11%) died due to massive bleeding. Of the four patients that required nonspecific intensive‐care intervention, all survived to the PICU admission.

## 4 Discussion

Our study describes the outcomes of a cohort of stem cell transplant patients from a single center in Colombia who required admission to the PICU over a 7‐year period. We found that 20% of children undergoing HSCT had at least one PICU admission, which is consistent with the broad range reported in other studies (see Table 3). Immunodeficiency was the most common underlying condition, reflecting a higher prevalence of pre‐transplant morbidities in this patient population. Recent studies have highlighted several adverse prognostic factors, including respiratory failure, multi‐organ failure, mechanical ventilation, and vasoactive agent support (Table 3). Our analysis confirms that invasive respiratory support (*p* = 0.02), higher VIS (*p* = 0.03), RRT (*p* = 0.04), and multi‐organ failure (*p* = 0.004) were significantly associated with poor outcomes.

**TABLE 3:** Summary of recent outcome in studies of children admitted to the pediatric intensive care unit after Hematopoietic stem cell transplant.

	Present study, Colombia	Szmit et al., 2020, Poland [4]	Zinter et al., 2020, USA [12]	Pillon et al., 2017, Italy [13]	An et al., 2016, China [14]	Zinter et al., 2015, USA [15]	Aspesberro et al., 2014, USA [16]
Years of study	2012–2019	2005–2017	2009–2014	1998–2015	2000–2012	2009–2012	2000–2006
Number of patients included in study	104	668	9183	496	302	1102	266
Age: Median (range)	7 years (1 month ‐ 15 years)	5.5 years (2.5 months‐19.7 years)	8 years (IQR 2–14)	8.3 years (0–23)	6 years (0.5–16)	9.1 years (±0.1)	6.95 years (0.5–18.9)
No PICU admissions *n* (%)	21 (20)	58 (8.7)	1532 (16.6)	70 (14.1)	29 (9.6)	288 (26)	53 (20)
Reasons for admission (%)	Respiratory failure (48%)	Respiratory failure (37.5%)	No information	Respiratory failure (36%)	Respiratory failure (62.07%)	No information	Respiratory problems
Mechanical ventilation *n* (%)	17 (68)	47 (81)	566 (37)	45 (64)	20 (68.97)	617 (34.6)	36 (14)
Ventilated survived *n* (%)	8 (47)	16 (34)	249 (44)	5 (11.1)	3 (15)	355 (57.5)	9 (25)
Most prominent risk factor associated with mortality	MV, higher inotropic score, RRT, presence of multi‐organ failure	Required MV, received more aggressive cardiac support, lower ANC on the last day of their PICU stay	Risk factors included AML, mismatched unrelated donor, mismatched umbilical cord blood allograft, admission to the PICU before day +100, and higher PRISM‐3 score.	Relapse, mismatched HSCT, lung or hepatic failure	Pediatric critical illness score	IPPV, RRT, ECMO, patients with viral or fungal infection	MV, RRT, cardiovascular support
Mortality (%)	43	58	16	65.7	72.4	16.2	50.9

Abbreviations: AML: acute myelogenous leukemia, ANC: absolute neutrophil count, ECMO: extracorporeal membrane oxygenation, HSCT: Hematopoietic stem cell transplant, IPPV: intermittent positive pressure ventilation, MV: mechanical ventilation, PICU: pediatric intensive care unit, RRT: renal replacement therapy.

The mortality rate among PICU patients in our cohort was similar to that of patients not admitted to the PICU, aligning with the declining mortality trend observed in recent studies (Table 3) [12]. Nevertheless, the low mortality rate in our cohort could be influenced by patients who received minimal interventions. Most PICU‐admitted HSCT patients (72%) required respiratory support, primarily invasive ventilation, and 68% needed support for two or more organ systems. Previous studies have shown that both invasive ventilation and multi‐organ failure are associated with higher mortality risk [4, 16, 17]. In our cohort, invasive ventilation was associated with increased mortality, with all non‐survivors requiring mechanical ventilation.

Respiratory failure was the leading cause of PICU admission, a finding consistent with previous studies (Table 3). Respiratory failure has been widely recognized as a major contributor to poor survival outcomes [14, 17, 18, 19, 20, 21]. Cardiovascular instability was present in 40% of PICU admissions, higher than 7.8% as reported by Szmit et al. [4]. However, the separate reporting of septic shock in their study may explain this discrepancy. HFOV was used early in the course of respiratory disease, primarily in cases of moderate to severe PARDS. In our cohort, HFOV was not associated with worse outcomes, consistent with other series where no higher mortality was found in patients requiring this modality [13, 16]. However, our results differ from those of Rowan et al., who reported higher mortality in HSCT patients with PARDS requiring HFOV [22]. Although the use of NIV might reduce the need for intubation, the small number of patients who received NIV in our study precludes definitive conclusions about its effect on mortality. The contrasting evidence between studies, such as Zinter et al.’s [15] findings of low mortality in HSCT patients requiring mechanical ventilation, suggests that mechanical ventilation may not necessarily increase mortality risk.

Infection, particularly septic shock was the leading cause of cardiovascular instability in our cohort. The majority of patients (60%) had a confirmed infection at the moment of PICU admission. Zinter et al. [15] reported a similar infection prevalence (45.7%) and noted a 22.2% mortality rate among infected patients. However, we found no significant difference in mortality between patients with and without infection (*p* = 0.69), suggesting that infection alone may not be the key determinant of outcomes in this population.

Cardiovascular support is an essential aspect of PICU management. In our cohort, patients receiving extensive cardiovascular support had a significantly lower chance of survival. The need for three or more cardiovascular agents, or a higher VIS was independently associated with poor prognosis. Although a high proportion of patients (84%) required cardiovascular support, 57% of those who experienced shock survived. This highlights the importance of early intervention and careful management of septic shock in this vulnerable population. Similarly, the use of RRT was associated with poor outcomes in our cohort, with only one of five RRT patients surviving their PICU stay. The late initiation of RRT in our cohort may explain this finding, as early RRT before MOF onset has been shown to improve outcomes in other studies [4, 13, 16, 23].

The PRISM score in our cohort was higher in the non‐survivors, but this difference did not reach statistical significance. Some studies have found no difference in PRISM scores between survivors and non‐survivors [2, 12, 16, 24], whereas others report higher scores in non‐survivors [25]. The use of PRISM scores in HSCT patients has been questioned, and it has been suggested that these scores should be adapted to incorporate HSCT‐specific factors [26]. Our hospital uses the first version of the PRISM score, which limits our ability to compare our findings with studies using more recent versions.

Haploidentical stem cell transplantation (haplo‐HSCT) with T‐cell replete grafts and post‐transplant cyclophosphamide (PT‐CY) is a rapidly growing approach to improve access to allogeneic HSCT, particularly in ethnic minorities. In our cohort, half of the patients underwent haplo‐HSCT, with no significant differences in mortality between haploidentical and other donor types. Although our findings suggest that patients undergoing haplo‐HSCT do not have worse outcomes when requiring PICU admission, prospective studies are needed to confirm these results.

The main limitation of this study is its retrospective single‐center design. Although the long study period provides valuable insights, the relatively small sample size limits the generalizability of our findings. Furthermore, the lack of more recent data may prevent the study from fully reflecting the impact of recent advancements in supportive care, such as improved ventilation strategies, novel infection management protocols, and immunotherapies. These advancements could significantly influence outcomes in critically ill HSCT patients, and future research should incorporate more current data and multicenter approaches to better understand the evolving landscape of pediatric HSCT care across different regions.

## 5 Conclusions

Children requiring PICU admission after Allo‐HSCT face high mortality rates, with invasive respiratory support, higher VIS, RRT, and MOF identified as key risk factors for poor outcomes. Despite improvements in supportive care, PICU admission remains associated with substantial risk. Future research should focus on multicenter prospective studies to validate these findings and explore standardized admission protocols and improved multidisciplinary collaboration between HSCT and PICU teams. We hope that continued research will further enhance outcomes and expand access to this potentially curative therapy for a broader range of patients.

## Author Contributions

All authors contributed to the study's conception and design. Material preparation and data collection were done by B.P., N.B., and L.N. Critical revision and analysis were done by L.N., B.P., and N.B. The first draft of the manuscript was performed by N.B., B.P., and L.N. All authors commented on previous versions of the manuscript and read and approved the final version to be published. In addition, agreement to be accountable for all aspects of the work and to ensure that questions related to the accuracy or integrity of any part of the work were appropriately investigated and resolved, was reached.

## Conflicts of Interest

The authors declare no conflicts of interest.

## Ethics Statement

The study was approved by the Institutional Ethics Committee of the Pablo Tobón Uribe hospital in accordance with the Declaration of Helsinki (approval number: 2018.074).

## Data Availability

The data that support the findings of this study are available on request from the corresponding author. The data are not publicly available due to privacy or ethical restrictions.
